# Endoscopic Findings in Patients With Uninvestigated Dyspepsia: A Retrospective Study From Qatar

**DOI:** 10.7759/cureus.11166

**Published:** 2020-10-26

**Authors:** Ahmad Badi, Vamanjore A Naushad, Nishan K Purayil, Prem Chandra, Hassan O Abuzaid, Firjith Paramba, Abdo Lutf, Mohamed Milad Abuhmaira, Abdel-Naser Y Elzouki

**Affiliations:** 1 Internal Medicine, Hamad Medical Corporation, Doha, QAT; 2 Medical Research, Hamad Medical Corporation, Doha, QAT; 3 Rheumatology, Hamad Medical Corporation, Doha, QAT; 4 Internal Medicine, Hamad Medical corporation, Doha, QAT

**Keywords:** endoscopy, dyspepsia, peptic ulcer disease, gastritis, helicobacter pylori

## Abstract

Background and objective

Dyspepsia is a common complaint encountered in general clinical practice. The prevalence of clinically significant endoscopy findings in dyspeptic subjects of various age groups and ethnicities in Qatar is not well studied. This study aimed to evaluate the prevalence of endoscopic findings in previously uninvestigated patients with dyspepsia.

Patients and methods

We retrospectively studied subjects older than 18 years of age who underwent endoscopy for dyspeptic complaints from January 2011 to December 2017. Subjects who already had peptic ulcer disease (PUD), those who underwent endoscopy for reasons other than dyspepsia, and those with incomplete data were excluded.

Results

A total of 824 subjects were reviewed for eligibility and 733 were included for analysis. The mean ±SD age of the study subjects was 42.7 ±13.5 years, and 59.5% of the subjects were male. Epigastric pain was the predominant symptom (79.2%) followed by heartburn (26.1%). Abnormal endoscopic findings were noted in 91.8% of subjects. Gastritis (65.5%) and oesophagitis (33.1%) were the most common findings observed. The overall prevalence of gastric ulcers was 4.6%, and it was higher in subjects who were more than 60 years of age (14.1%, p=0.001). Gastric carcinoma was seen in only four (0.54%) subjects.

Conclusion

Gastritis was the most common endoscopic finding observed followed by oesophagitis. The most common presenting symptoms were epigastric pain and heartburn. The prevalence of gastric ulcers was significantly high in patients above 60 years of age, and the incidence of gastric carcinoma was low in the study population.

## Introduction

Dyspepsia is a common but nonspecific complaint in adults seeking medical consultation. In primary care settings, dyspepsia accounts for 2-5% of all consultation among adults [1], and up to 40% of the general population reports symptoms related to dyspepsia [2,3]. The exact prevalence of dyspepsia is not known and it varies with gender and the country of origin. The National Institute for Health and Care Excellence (NICE) guidelines describe dyspepsia as a range of symptoms that include upper abdominal pain or discomfort, heartburn, gastric reflux, nausea, or vomiting [4]. The Rome IV criteria define dyspepsia as any combination of the following four symptoms: postprandial fullness, early satiety, epigastric pain, and epigastric burning, which are severe enough to interfere with the usual activities and occur at least three days per week over the past three months with an onset of at least six months in advance [5]. Several diseases present with the above symptoms, which include peptic ulcer disease (PUD), inflammatory bowel disease (IBD), and malignancy. In addition, dyspeptic symptoms may also result from a variety of other conditions such as pancreatitis, biliary tract disease, and acute coronary syndrome.

Dyspepsia is not only a common symptom but may also be a marker of an underlying structural disease. Malignancy is present in 1-3% of patients with dyspepsia and PUD is seen in 5-15% of patients [6-8]. Given the wide range of presentations, it is a tendency among clinicians to treat it empirically with acid suppression therapy in the early stages, especially in younger age groups. The role of barium studies in evaluating patients with dyspepsia has low sensitivity and specificity compared with upper gastrointestinal endoscopy (endoscopy), and it does not allow obtaining a biopsy specimen. Therefore, the utility of a barium study should be generally limited to patients for whom endoscopy is risky. Although many guidelines have been published on dyspepsia, much controversy still exists regarding the most appropriate management strategy. The past medical guidelines on the indication for early endoscopy in patients with dyspepsia were based on age and alarm features. The guidelines recommended an early endoscopy in patients with alarm features regardless of their age and for patients with new-onset dyspepsia after 45-55 years of age, while in younger patients without alarm features, either empirical proton pump inhibitors (PPI) or *Helicobacter pylori (H. pylori)* test and treat were the initial treatment strategies [6,9-14]. Despite having multiple guidelines on the role of endoscopy in dyspepsia, several studies have shown that alarm features have limited predictive value for an underlying malignancy [8,15-17]. The updated 2017 American College of Gastroenterology (ACG) and the Canadian Association of Gastroenterology (CAG) guidelines recommend that patients ≥60 years of age presenting with dyspepsia be investigated with endoscopy to exclude organic pathology, whereas patients at a higher risk of malignancy, such as those who had spent their childhood in a high-risk gastric cancer country or those with a positive family history, could be offered an endoscopy at a younger age [18]. Alarm features should not automatically prompt endoscopy in younger patients but this should be considered on a case-by-case basis [18].

The aim of the present study was to evaluate the prevalence of abnormal endoscopic findings in previously uninvestigated patients with dyspepsia who underwent an endoscopy and to study the distribution of these abnormal findings among various age groups and nationalities.

## Materials and methods

Study design and study subjects

A cross-sectional retrospective study was conducted at Al Khor Hospital, Hamad Medical Corporation, Qatar. All subjects older than 18 years of age who underwent endoscopy for dyspeptic complaints from January 2011 to December 2017 were included in the study. Dyspepsia was defined according to NICE guidelines, which describe dyspepsia as a range of symptoms that include upper abdominal pain or discomfort, heartburn, gastric reflux, nausea, or vomiting [4]. Regarding subjects who had undergone more than one endoscopy during the study period, the first endoscopy was taken as the index endoscopy. Subjects who already had PUD, those who underwent endoscopy for reasons other than dyspepsia, and those with incomplete data were excluded.

Data collection

Data were retrieved from the medical records file and electronic database using the healthcare numbers. This included basic demographics, comorbid conditions, symptoms and signs, and information related to smoking, alcohol intake, and medication use. The endoscopic data included sites and types of abnormality, biopsy results (if available), and results of the Campylobacter-like organism (CLO) test (rapid Urease test) for *H. pylori*.

Statistical analysis

Descriptive statistics were used to describe the data. For continuous variables, the mean and standard deviations (SD) were used to summarize the data. For categorical variables, frequencies and percentages were reported. The differences between groups were analyzed using Pearson's chi-squared test or Fisher's exact test. A p-value of <0.05 was considered statistically significant. The data were processed and analyzed using SPSS Statistics version 22 (IBM, Armonk, NY).

## Results

Demographic characteristics

A total of 824 subjects were enrolled, of which 91 were excluded (incomplete information=53, repeat endoscopy=15, non-dyspeptic causes=23), leaving a final cohort of 733 for analysis. The mean age ±SD of the study subjects was 42.71 ±13.5 years, and 59.5% of the subjects were male. Diabetes (14.9%) and hypertension (13.1%) were the most common comorbid conditions observed. A total of 49 subjects were smokers and 23 were consuming alcohol. Data on smoking and alcohol were not available for 299 and 308 subjects respectively. Epigastric pain was the predominant symptom (79%) followed by heartburn (26.1%) in the overall cohort. Baseline demographic features and presenting symptoms at the time of endoscopy are shown in Table 1.

Prevalence of positive findings on endoscopy

Clinically significant endoscopic findings were found in 91.8% of study subjects. The proportion of subjects with significant findings in the oesophagus, stomach, and duodenum were 73.5%, 33.3%, and 14%, respectively. On sub-analyzing this data, oesophagitis was found to be the most common oesophageal abnormality (33.1%). Gastritis was found to be the most common abnormality in the stomach (65.5%), and duodenitis was the most common endoscopic duodenal finding (11.7%). Gastric and duodenal ulcers were seen in 4.6% and 5.9% of subjects, respectively. Gastric carcinoma was seen in only four (0.54%) patients (Table 2). The CLO test for *H. pylori *infection was positive in 35.6% of the study subjects.

Presenting symptoms and endoscopic findings according to age groups

For analytical purposes, the subjects were categorized into three groups according to age: <40 years, 40-59 years, and ≥60 years. An analysis of the subgroups revealed that the least number of subjects were in ≥60 years group (9.8%); however, gender distribution across all the three groups was similar (p=0.943). Similar to the results in the overall cohort, epigastric pain and heartburn were the predominant symptoms across the three age groups. Melena was more common in the older age group (Table 1).

**Table 1:** Distribution of demographics features and symptoms CAD: coronary artery disease; CKD: chronic kidney disease

Variable	Total patients	<40 years	40-59 years		≥60 years	P-value
	N=733	N=327 (44.6%)	N=335 (45.7%)	N=71 (9.8%)		
Gender	N (%)	N (%)	N (%)	N (%)		0.943
Male	437 (59.5)	196 (59.9)	199 (59.4)	41 (57.7)		
Female	296 (40.5)	131 (40.1)	136 (40.6)	30 (42.3)		
Nationality						<0.0001
Qatar	196 (26.6)	94 (28.7)	79 (23.4)	23 (32.4)		
Asia	247 (33.7)	111 (33.9)	126 (37.7)	10 (14.1)		
Middle East (excluding Qatar)	128 (17.5)	61 (18.7)	44 (13.2)	23 (32.4)		
Africa	148 (20.2)	58 (17.7)	76 (22.8)	14 (19.7)		
Others	14 (1.9)	3 (0.9)	10 (3.0)	1 (1.4)		
Comorbidity						
Hypertension	102 (13.9)	9 (2.8)	54 (16.2)	39 (54.9)		< 0.001
Diabetes	96 (13.1)	7 (2.1)	49 (14.7)	40 (56.3)		<0.001
Asthma	20 (2.7)	7 (2.1)	12 (3.6)	1 (1.4)		0.401
CAD	19 (2.6)	1 (0.3)	7 (2.1)	11 (15.5)		<0.001
CKD	11 (1.5)	1 (0.3)	6 (1.8)	4 (5.6)		0.003
Liver Disease	22 (3)	3 (0.9)	16 (4.8)	3 (4.2)		0.012
Symptoms						
Epigastric pain	580 (79.2)	266 (81.3)	264 (79)	50 (70.4)		0.12
Belching	62 (8.5)	37 (11.3)	19 (5.7)	6 (8.5)		0.34
Nausea	78 (10.7)	43 (13.1)	29 (8.7)	6 (8.5)		0.148
Vomiting	69 (9.4)	32 (9.8)	29 (8.7)	8 (11.3)		0.76
Heartburn	191 (26.1)	86 (26.3)	89 (26.6)	16 (22.5)		0.769
Early satiety	21 (2.9)	12 (3.7)	6 (1.8)	3 (4.2)		0.273
Melena	50 (6.8)	20 (6.1)	22 (6.6)	8 (11.3)		0.288
Diarrhea	6 (0.8)	5 (1.5)	1 (0.3)	0		0.155

The total prevalence of clinically significant findings on endoscopy was similar in all the three age groups. On analyzing the overall disease-specific pattern of involvement in each age group, a similar pattern of abnormality was seen; that is, gastritis was the most common finding followed by oesophagitis. The prevalence of site-specific findings was also similar in all the three age groups with the stomach being the most commonly involved site (69.4%, 76.3%, 78.95%; in <40 years, 40-59 years, and ≥60 years, respectively, p=0.073), while duodenum was found to be the least involved site (14.1%, 14.7%, 11.3%; p=0.755) (Table 2).

**Table 2:** Endoscopic findings among various age groups In cases where percentage values do not add up to 100%, each subject may have more than one endoscopic finding

	Total patients=733 (100%); n (%)	<40 years; n=327 (44.6%); n (%)	40-59 years; n=335 (45.7%); n (%)	≥60 years; n=71 (9.8%); n (%)	P-value
Oesophagus					
Oesophagitis	243 (33.15)	110 (33.6)	115 (34.32)	18 (25.35)	0.652
Oesophageal varices	18 (2.5)	3 (0.9)	11 (3.3)	4 (5.6)	0.027
Hiatus hernia	11 (1.5)	6 (1.8)	4 (1.2)	1 (1.4)	0.795
Stomach					
Gastritis	481 (65.5)	202 (61.7)	231 (68.9)	48 (67.6)	0.233
Ulcer	34 (4.6)	10 (3.1)	14 (4.2)	10 (14.1)	<0.001
Fundal varices	4 (0.5)	0	2 (0.6)	2 (2.8)	0.014
Carcinoma	4 (0.5)	0	3 (0.9)	1 (1.4)	0.171
Duodenum					
Erosive duodenitis	86 (11.7)	38 (11.6)	41 (12.3)	7 (9.9)	0.844
Ulcer	43 (5.9)	16 (4.9)	24 (7.2)	3 (4.2)	0.376
Normal endoscopy	60 (7.2)	27 (8.3)	26 (7.8)	7 (8.9)	0.563

Oesophagus

In oesophagus, oesophagitis was the most common finding seen across all three age groups (33.6%, 34.32%, 25.35%; p=0.652). There was no statistically significant difference in the prevalence of various endoscopic oesophageal findings between the groups.

Stomach

A high prevalence of gastritis was observed in all three groups (61.7%, 69.1%, and 67.6%), but the difference was not statistically significant (p=0.233). The prevalence of gastric ulcers was significantly higher in subjects above 60 years. Only four subjects had gastric carcinoma, of which three were in the age group of 40-59 years.

Duodenum

Erosive duodenitis was the most common duodenal finding noted in all the groups. Duodenal ulcer was more common in the age group of 40-59 years (7.2%) when compared to the other two age groups, but the difference was not statistically significant (p=0.376).

Endoscopic findings according to ethnicity

When endoscopic findings were compared across various ethnic groups, there was no statistically significant difference among them (Table 3).

**Table 3:** Distribution of endoscopic findings in various ethnic groups

	Qatar (n=196)	Asia (n=247)	Middle East excluding Qatar (n=128)	Africa (n=148)	Others (n=14)	P-value
Total	179/196 (91.3%)	226/247 (91.5%)	118/128 (92.2%)	137/148 (92.6%)	12/14 (85.7%)	0.795
<40 years	86/94 (91.5%)	102/111 (91.9%)	55/61 (90.2%)	55/58 (94.8%)	2/3 (66.6%)	0.032
40-59 years	72/79 (92.3%)	115/126 (91.3%)	41/44 (93.2%)	71/76 (93.4%)	9/10 (90%)	0.978
≥60 years	21/23 (91.3%)	9/10 (90%)	22/23 (95.7%)	11/14 (78.6%)	1/1 (100%)	0.551

## Discussion

The results of this study showed a high prevalence of significant endoscopic findings in subjects with dyspepsia. Significant endoscopic findings were observed in 91.8% of study subjects, which is higher than the previously reported rates [15,19-23]. A study done by Thomson et al. reported 58% of patients having significant findings on endoscopy [24]. The higher prevalence rate in our study carries more significance than the study done by Thomson et al. due to the fact that they excluded patients with acid suppression therapy or prokinetic therapy two to four weeks prior to the study. Another study done on dyspeptic patients unresponsive to PPIs undergoing endoscopy found abnormal findings in around 67% of patients. They included only patients between the ages of 18 to 45 years [22].

We observed that epigastric pain was the most common presenting symptom (79.2%) in the overall study population, which is consistent with the findings of a previous study by Khaled et al., who reported 76.6% having epigastric pain as the most common presenting symptom [20], while it was 34% in another study [24]. Our study revealed a higher prevalence of gastritis compared to other diseases (65.5%), which is higher than the reported rates in the past [20,22,24].

The overall prevalence of malignancy was found to be low in the present study subjects (0.54%), and all subjects with malignancy were above 40 years of age. This is in partial agreement with the results of a past study done by Thomson et al. where they found malignancy in only two patients and both were above 50 years of age [24]. Similarly, other studies have also reported a low prevalence of malignancy (0.8%) [20]. In our study, the prevalence rates of gastric and duodenal ulcers were found to be 4.6% and 5.9%, respectively. Previous studies have reported similar rates for gastric ulcers while the prevalence of duodenal ulcers showed conflicting results [20,22]. Mestan et al. have reported the prevalence of gastric ulcer as 3.6% and that of duodenal ulcer as 8.1%, whereas in another study, it was found to be 2.6% and 1.7%, respectively [20,22].

We also examined the presence of significant endoscopic findings among different nationalities, and it was found to be high in all the ethnic groups; however, the difference among them was not statistically significant. On a sub-analysis of subjects of various ethnicities according to age groups, a similar pattern of involvement was observed. Since the state of Qatar has a large number of expatriate population from across the globe, this observation carries significance. A previous study from Qatar showed that 80% of the study population had abnormal endoscopic findings. Their study population included two groups: Qatari nationals with a mean age of 37 years and expatriates with a mean age of 38 years. They did not find any significant difference in endoscopic findings between the two groups [25].

There are a few other studies done in the Middle East region on the prevalence of PUD in the past. Two studies from Saudi Arabia have reported a prevalence of 21.9% and 21.2% respectively. The first study, which had a study population mainly between 18-25 years of age, showed a high prevalence of gastric ulcer (16.2%) and duodenal ulcer (5.6%). It found that PUD was more common in the younger age group of 18-45 years when compared to patients above 45 years of age [26]. Another study done among the elderly population in Saudi Arabia showed a PUD prevalence of 21.2% [27] and found that it was more common in females [22].

Studies in other parts of Asia have shown a higher prevalence of PUD compared to the European population. A study from China [28] has reported a prevalence of 17.2% (gastric ulcer 6.1% versus 13.3% for duodenal ulcer), whereas studies in Europe have shown a lower prevalence between 4.1-6.2% [29]. The higher prevalence in China could be due to a high prevalence of *H. pylori* infection among the country's population [28]. A study conducted in Hong Kong has reported that 60.9% of patients with PUD were in the age group of 60-70 years [30], whereas another study has shown that individuals in the age group of 40-49 years were significantly more likely to have PUD compared to those between 30-39 years of age [29].

Limitations

The present study has some limitations. Firstly, a correlation of risk factors like smoking and medications with the occurrence of significant endoscopic findings was not done because the specific details were not available for all the study subjects. Secondly, we did not correlate between *H Pylori* infection and endoscopic findings, which might have precluded us from inferring the reason for the high prevalence of significant endoscopic findings.

A preprint of this manuscript was published and is available in the link provided below. However, it is not peer-reviewed or published in any other journal. https://www.researchsquare.com/article/rs-30323/v1. DOI:10.21203/rs.3.rs-30323/v1

## Conclusions

Our study found that the prevalence of abnormal endoscopic findings was high in subjects presenting with dyspepsia across all age groups and nationalities. Gastritis was the most common endoscopic finding observed followed by oesophagitis. The most common presenting symptoms were epigastric pain and heartburn. The prevalence of gastric ulcers was significantly high in patients older than 60 years, and the prevalence of gastric carcinoma was low in the study population.
